# Automated calculation of coagulation activity and International Normalized Ratio in oral anticoagulant control

**DOI:** 10.1155/S1463924687000373

**Published:** 1987

**Authors:** R. H. M. Peters, A. C. T. H. Gehring, F. M. F. G. Olthuis

**Affiliations:** Department of Clinical Chemistry Hospital ‘De Stadsmaten’ Ariënsplein 1 Enschede 7511 JX The Netherlands


Journal of Automatic Chemistry/*Journal of Clinical Laboratory Automation*, Vol. 9, No. 4 (October–December 1987), pp. 189–193

### Automated calculation of coagulation activity and International Normalized Ratio in oral anticoagulant control

**R. H. M. Peters, A. C. T. H. Gehring and F. M. F. G. Olthuis***

*Department of Clinical Chemistry, Hospital 'De Stadsmaten' Ariënsplein 1, 7511 JX Enschede, The Netherlands*

*Thromboplastin reagents are used to perform the prothrombin time test in the laboratory control of oral anticoagulant therapy. Previously the measured clotting time was transformed to activity either by reading the value from a correlation curve or from a table supplied by the reagent's manufacturer. An interface which connects the coagulometer to a personal computer, and software to handle the clerical work, has been developed by the authors. Up to 12 channels can be analysed simultaneously with this system. The results are calculated on-line and can be reported as activity, as well as by the WHO's International Normalized Ratio. Because of the reduction in clerical work, a time-saving of 35% was achieved in the authors' laboratory; additionally, the chance of making mistakes is reduced.*

### Introduction

In the laboratory control of oral anticoagulant therapy the prothrombin time (PT) test is the first test of choice. Most methods for measuring PT are based on the measurement of the clotting time, in seconds, of a sample. Instruments have been developed for automatic measurement of PT, based on electrical or photo-optical detection of clotting. For clinical use different methods of expressing the PT result, for example seconds, ratio, index or activity, are used. Moreover, thromboplastin reagents differ greatly in response to the degree of anticoagulation or clotting factor deficiency. Because of the normalization of the PT in oral anticoagulant control the World Health Organization (WHO) developed a system of thromboplastin calibration, and introduced the International Normalized Ratio (INR) as a common scale for reporting the PT result [1]. Whether expressing the PT result as a ratio, index, activity or INR, the primary result in seconds has to be transformed to the unit used, by calculation or by reading the value from a correlation curve or table. This transformation can be easily carried out by a computer connected to the coagulometer, thereby reducing the time of analysis and the chance of making mistakes.

Four years ago the authors developed an interface to connect a coagulometer to an Apple IIe microcomputer (Apple Computer, Inc., Cupertino, California, USA). Software was written in Applesoft Basic to handle the clerical work [2]. This system has been in operation for three years without any technical failure. Later it was decided to use the IBM PC to connect laboratory analysers to the hospital's mainframe computer. Because the interface for the coagulometer had been designed specifically for an Apple II computer, an interface was developed according to the commonly used RS232 protocol. The computer program was rewritten in Pascal. In this paper the way of calculating the coagulation activity and the INR, as well as some features of the computer program, are described.

* Correspondence to Dr Olthuis.

### Materials ana methods

*Apparatus*

The LC-6 coagulometer (Lode Instruments, Groningen, The Netherlands) is a six-channel coagulometer, which detects clot formation photo-optically. The coagulometer is equipped with a pipette and the start signal is remotely controlled. An IBM PC-XT microcomputer (operating system DOS 3.1), equipped with 640 kB random access memory, two disk-drives (360 kB), an asynchronous communication adapter, and a parallel adapter (International Business Machines Corporation, Boca Raton, Florida, USA) (see figure 1). An IRMA 3278/79 Terminal Emulator Card (Digital Communications Associates Inc., Alpharetta, Georgia, USA) was used to link the IBM PC-XT on-line to the hospital's mainframe (an IBM 4361), which contains the patient data-base. For printing Epson FX85 printer (Seiko Epson Corporation, Nagano, Japan) was used.

An interface (RS 232) was developed to connect the LC-6 to a microcomputer (see Appendix). This interface was built in collaboration with Lode Instruments and was built into the LC-6. The interface was designed to allow a second LC-6 to be connected to the interface. The computer program was written in Pascal (IBM Pascal compiler, version 2.0).

*Reagents*

Thrombotest TM thromboplastin reagent was purchased from Nycomed & Co. (Oslo, Norway).

### Calculations

*Expression of the PT result as activity*

Loeliger *et al.* [3] observed for the Thrombotest reagent a linear relationship between the clotting time ratio (patients' time/normal time) and $100/c$, in which $c$ is the mean concentration of the clotting factors II and X in percentage of normal. As is shown in figure 2, a linear

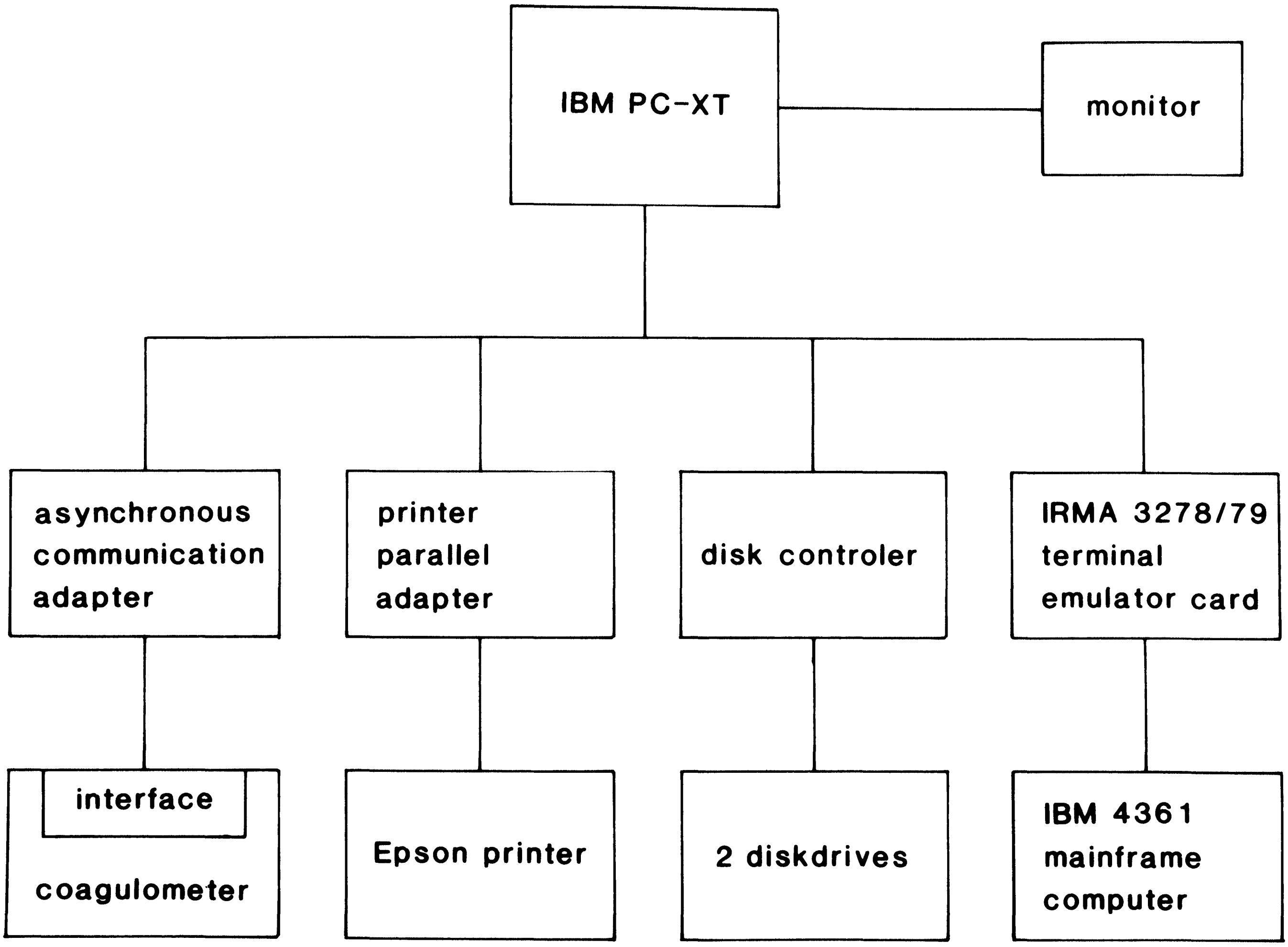

*Figure 1. Schematic diagram of the computerized coagulometer.*

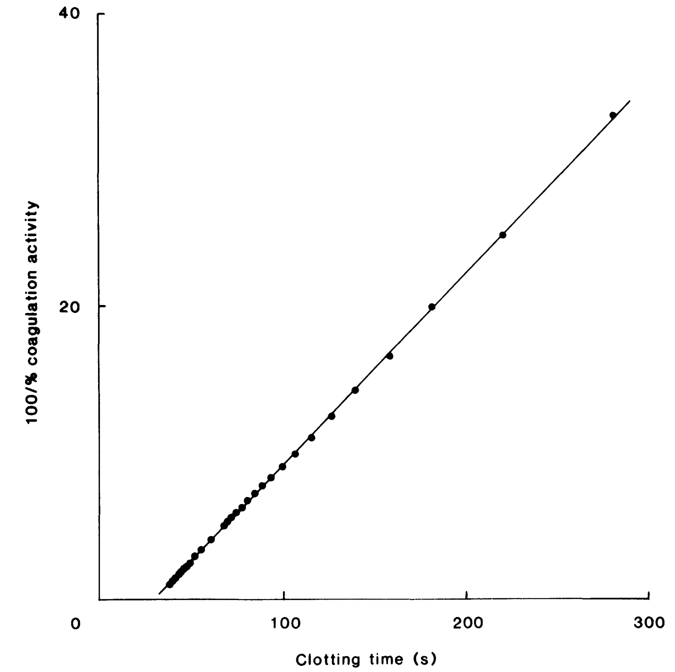

*Figure 2. Relation between % coagulation activity and clotting time for the thrombotest reagent batch No. 916.*

relationship exists between the clotting time and 100/ % coagulation activity, where the coagulation activity is read from the correlation table supplied by the manufacturer. This principle is used to calculate the coagulation activity: for every new batch of thrombotest reagent the linear regression line of the clotting times and 100/ % coagulation activity is calculated. With the calculated slope and intercept the coagulation activity of the patient samples is calculated from the measured clotting time by the equation: Coagulation activity(%) =

$$^{100}/_{(\text{clotting time} \times \text{slope} + \text{intercept})}.$$

*Expression of the PT result as INR*

INR is calculated with the following equation:
INR = $(PT_{patient}/PT_{normal})^{ISI}$ in which $PT_{patient}$ is the measured prothrombin time in s, $PT_{normal}$ the mean normal prothrombin time in s and ISI the International Sensitivity Index [4]. The ISI is supplied by the manufacturer and is specific for one batch of thromboplastin reagent. $PT_{normal}$ has to be measured by the laboratory itself and is specific for a batch of thromboplastin and the method used.

### Results

To investigate the method of calculating the percentage coagulation activity, the calculated coagulation activity was compared with that read from the correlation cable in

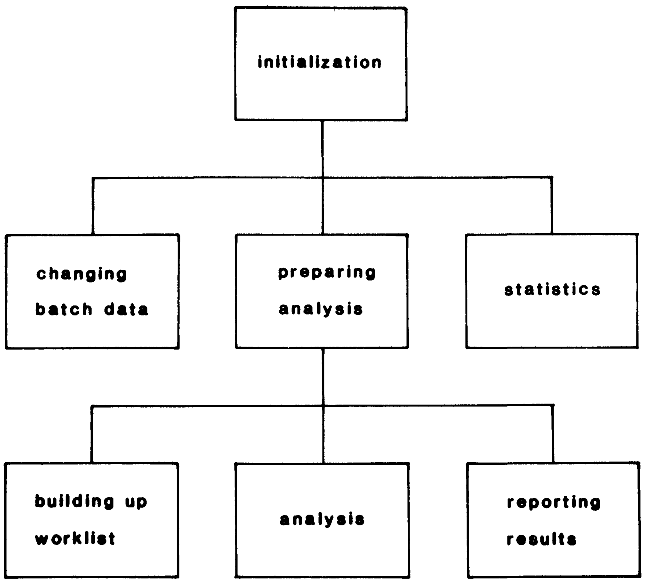

*Figure 3. Schematic diagram of the main modules of the program.*

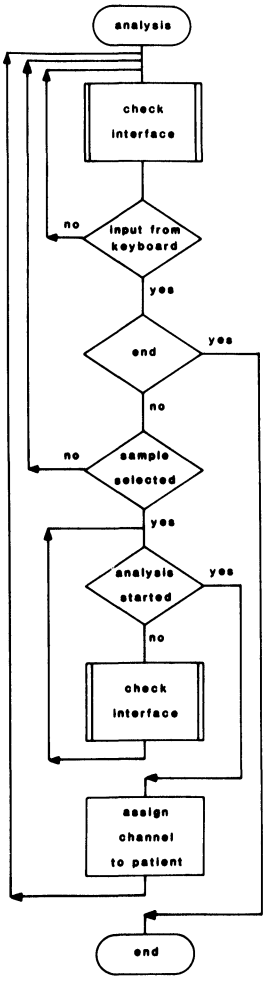

(*a*)

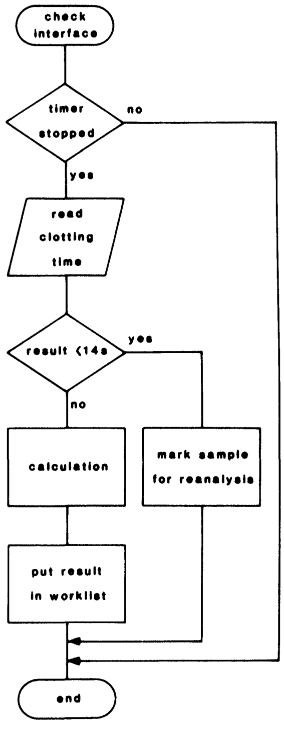

(*b*)

*Figure 4(a). Flowchart of the subprogram 'analysis'.*

*Figure 4(b). Flowchart of the subroutine 'check interface'.*

the package insert for 13 different batches of thrombotest reagent. The regression lines of the clotting times and 100/ % coagulation activity were calculated for four different sets of data per batch:

(1) All of the 30 data-points supplied by the manufacturer.

(2) The data-points of 100, 60, 30, and 15% activity.

(3) The data-points of 80, 40, 20, and 10% activity.

(4) The data-points of 40, 20, 10, and 5% activity.

With the calculated slope and intercept for each data-set the percentage coagulation activity was calculated from the clotting time values of the 30 data-points read from the correlation table. The coagulation activities calculated with data-set (1) and (3) did not differ significantly (Wilcoxons' Signed Rank Test) from the coagulation activities read from the correlation table for all the 13 batches tested. However, when the activities were calculated with data-set (2) and (4), three ($p < 0{\cdot}001$) respectively, two ($p < 0{\cdot}01$) batches differed significantly. Because the results with the four-point regression line of data-set (3) were as good as that with the 30-point regression line of data-set (1) data-set (3) was used in the program to calculate the coagulation activity.

*Brief description of the computer program*

The main modules of the program are shown in figure 3 and will be described below.

*Initialization:* the devices (printer, asynchronous communication adapter and IRMA card) will be initialized and the batch data are loaded from disk into memory.

*Changing batch data:* when a new batch of thrombotest reagent is used, the data concerning that batch have to be entered into the microcomputer through the keyboard as follows:

(1) Batch number.

(2) The clotting times corresponding to 80, 40, 20 and 10% coagulation activity, respectively. From this data the regression parameters slope and intercept are calculated automatically.

(3) The ISI and the mean normal PT, to compute the INR. These data are stored on disk. In this part of the program one can define whether the samples should be analysed singly or in duplicate, and whether the result should be reported in percentage activity, INR or both.

*Preparing analysis:* a previously compiled work-list is loaded from disk into memory.

*Building up the work-list:* to create the work-list such patient information as sample identification number, name and date of birth can be entered in the computer through the keyboard or can be downloaded from the mainframe by the PC – mainframe link. Depending on the lay-out of the work-list, the information and test results of at least 750 patients can be held in memory at any one time.

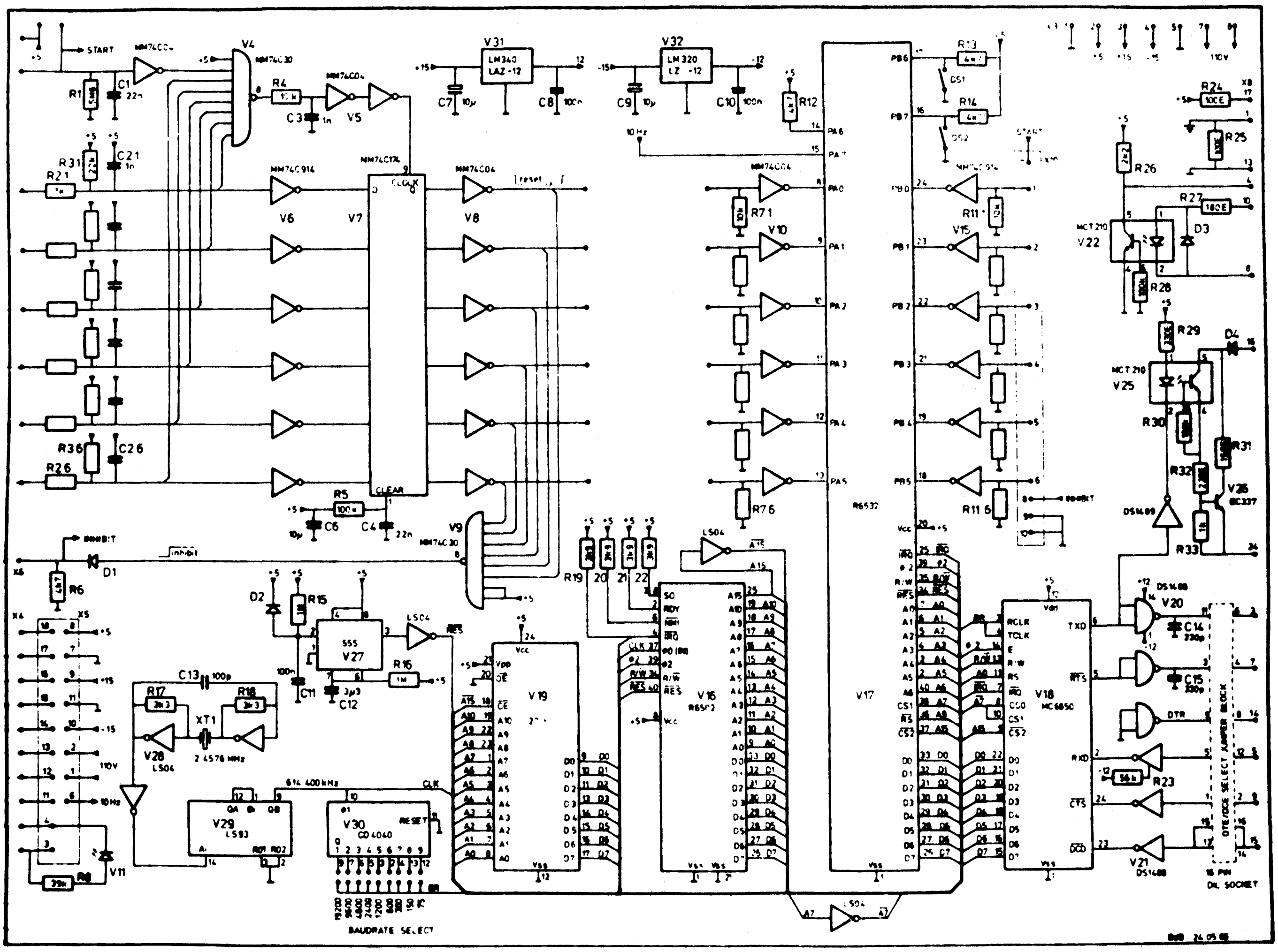

*Figure 5. Diagram of the electronic part of the interface.*

*Analysis:* the samples can be either randomly analysed or analysed in ascending order of sample identification number. The operator selects a sample for analysis and starts the determination by pipetting the citrate blood in the tube with preheated thrombotest reagent. The program checks which channel of the coagulometer is started and the next sample can be selected (see figure 4[*a*]). The timer of a channel stops when a clot is formed and the result is sent to the microcomputer (figure 4[*b*]). The resolution of the clotting time is 0·1 s. If the clotting time is less then 14·0 s, the result is rejected, because the timer has stopped by other reasons than clot formation. The sample has to be reanalysed. From the accepted results, the percentage coagulation activity and/or the INR is calculated from the clotting time. Every 15 samples the results are stored on disk. Samples can be analysed in duplicate; when the difference between duplicate clotting times is less than by the user predefined percentage, the mean is calculated.

*Reporting results:* the results, expressed as activity, INR or both, can either be sent to the mainframe, or can be printed out selected by ward or by patient identification number.

*Statistics:* for quality control up to five different control samples can be analysed; the results are stored on disk. Means, standard deviations and coefficient of variations can be calculated and printed on request.

### Discussion

In most laboratories the percentage coagulation activity is determined from the measured clotting time by reading the value from a correlation curve or table. With the present method the coagulation activity (as a percentage) can be calculated automatically. The computed coagulation activities are comparable with that read from the correlation table supplied by the manufacturer in the whole range of activities from 3 to 100%, when the regression line was calculated from the clotting times corresponding with 80, 40, 20, and 10% activity and 100/ % coagulation activity. We also found a linear relationship between 100/ % coagulation activity and the clotting time for the PT reagent Normotest (Nycomed, Oslo, Norway), which is used to evaluate haemostatic function, for example in preoperative screening or in patients with liver disease.

Several studies have shown that the instruments used for automated measurement of the prothrombin time have a significant effect on the result. Some of the bias due to different instruments is eliminated by the use of ratios [5]. The INR is calculated from the ratio of the patients'

prothrombin time to the mean normal prothrombin time and the ISI. Until now, the INR for each patient has had to be calculated, or a correlation table has had to be prepared to avoid the need to use a calculator.

With the described system of a coagulometer connected to a microcomputer by the interface and the computer program, the clotting time is transformed automatically to percentage coagulation activity and/or INR. The microcomputer controls which of the six channels (or 12 when two coagulometers are used) is in use for a particular patient sample, and the result is calculated on-line and stored on disk. Because of the reduction in clerical work, the chance of making mistakes, as well as the time of analysis, is reduced. In our laboratory about a 35% time-saving was achieved.

The present system of interface and software is also applicable to other coagulometers.

With the developed computer program it is easy to report the Thrombotest result as activity, as well as INR. This double reporting system, as recommended by Loeliger *et al.* [6], will hold to introduce the INR calibration system to clinicians and general practitioners.

### References

1. WHO Expert Committee on Biological Standardization, *Technical Report Series 687* (WHO, Geneva 1983), p. 81.
2. Peters, R. H. M. and Olthuis, F. M. F. G., Abstract in the proceedings of the 3rd symposium on *Automatisering in Medische Laboratoria* (Groningen, 1985).
3. Loeliger, E. A., Meuwisse-Braun, J. B., Muis, H., Buitendijk, F. J. J., Veltkamp, J. J. and Hemker, H. C., *Thrombosis et Diathesis Haemorrhagica*, **23** (1970), 569.
4. International Committee for Standardization in Haematology/International Committee on Thrombosis and Haemostasis, *Thrombosis and Haemostasis*, **53** (1985), 155.
5. Van den Besselaar, A. M. H. P., *Haemostasis*, **15** (1985), 271.
6. Loeliger, E. A., Poller, L., Samama, M., Thomson, J. M., Van den Besselaar, A. M. H. P., Vermylen, J. and Verstraete, M., *Thrombosis and Haemostasis*, **54** (1985), 515.

### Appendix: Brief description of the interface

The electronic components of the interface are shown in figure 5. The interface's microprocessor checks the status of the channels of the coagulometer 10 times a second: when the timer of a channel is running the status is 1, otherwise it is 0. Using the change in status the microprocessor calculates the clotting time. The personal computer checks the status of each channel by sending a code to the interface. The interface responds by sending the status to the PC. When a timer of the coagulometer is stopped, the status is changed and the PC sends a code specific for that channel to the interface. The interface responds by sending the clotting time of that channel to the PC.

**SOME 39th PITTSBURGH SYMPOSIA**

*22–26 February 1988, New Orleans, USA*

Alternatives in atomic spectrometry *arranged by G. M. Hieftje, Indiana University*

Analysis of high $T_c$ superconducting oxides *arranged by W. A. Byers, Westinghouse Research and Development*

Thirty-year anniversary of capillary (Gas) chromatography *arranged by L. S. Ettre, Perkin-Elmer*

Flow injection analysis in process control and biotechnology *arranged by J. F. Coetzee, University of Pittsburgh and J. Ruzicka, University of Washington*

Future potential of microcolumn separation techniques *arranged by M. L. Lee, Brigham Young University*

Seeing atoms: building, buying and using scanning tunneling microscopes *arranged by B. J. Bulkin and D. A. Chernoff, Standard Oil Research and Development*

Spectroscopic investigation of antiques *arranged by G. L. Vassilaros, Crucible Materials Corporation*

Field-flow fractionation *arranged by J. C. Giddings, University of Utah*

Near, infrared, spectroscopy *arranged by E. W. Starck, KES Analysis*

Capillary zone electrophoresis *arranged by A. G. Ewing, Pennsylvania State University*

MS/MS in analytical chemistry: trends and forecasts *arranged by K. L. Busch, Indiana University*

Mass spectrometry of large molecules *arranged by F. W. McLafferty, Cornell University and A. Benninghoven, University of Münster*

On-site environmental analysis *arranged by S. L. Shockey, Center for Hazardous Materials Research, and K. R. Martin, PENN-RUN Corporation*

Energy dispersive X-ray fluorescence spectroscopy *arranged by F. W. Kunz, Ford Motor Company*

Advances in Raman spectroscopy *arranged by S. A. Asher, University of Pittsburgh*

ASTM E-42: probing chemistry at surfaces *arranged by R. W. Linton, University of North Carolina*

Drug testing in the workplace *arranged by H. S. Hertz, National Bureau of Standards*

Artificial intelligence applications in analytical chemistry *arranged by S. H. Peterson, Westinghouse Research & Development Center*

Microelectronic chemical sensors *arranged by R. E. Dessy, Virginia Polytechnic Institute and State University*

Organizing and managing the analytical laboratory *arranged by J. G. Grasselli, Standard Oil Research & Development*

User-manufacturer information exchange symposia (UMIX) *arranged by Robin L. Garrell, University of Pittsburgh*

*Details from Pittsburgh Conference, Dept. UPD, 12 Federal Drive, Suite 322, Pittsburgh, PA 15235, USA.*

